# [*N*,*N*-Bis(diphenyl­phosphino)isopropyl­amine]dibromidonickel(II)

**DOI:** 10.1107/S1600536809003936

**Published:** 2009-02-06

**Authors:** Marko Hapke, Anina Wöhl, Stephan Peitz, Bernd H. Müller, Anke Spannenberg, Uwe Rosenthal

**Affiliations:** a Leibniz-Institut für Katalyse e. V. an der Universität Rostock, Albert-Einstein-Strasse 29a, 18059 Rostock, Germany; b Linde AG, Linde Engineering Division, Dr.-Carl-von-Linde-Strasse 6-14, 82049 Pullach, Germany

## Abstract

The title compound, [NiBr_2_(C_27_H_27_NP_2_)], was synthesized by the reaction of NiBr_2_(dme) (dme is 1,2-dimethoxy­ethane) with *N*,*N*-bis­(diphenyl­phosphino)isopropyl­amine in methanol/tetra­hydro­furan. The nickel(II) center is coordinated by two P atoms of the chelating *PNP* ligand, Ph_2_PN(*i*Pr)PPh_2_, and two bromide ions in a distorted square-planar geometry.

## Related literature

For derivatives of the title compound and their structural details, see: Cooley *et al.* (2001 ▶); Sushev *et al.* (2005 ▶); Sun *et al.* (2006 ▶). For structural features of a nickel complex with an arene-briged bis-*PNP* ligand, see: Majoumo-Mbe *et al.* (2005 ▶). For catalytic features of the *PNP* ligand, see: Wöhl *et al.* (2009 ▶).
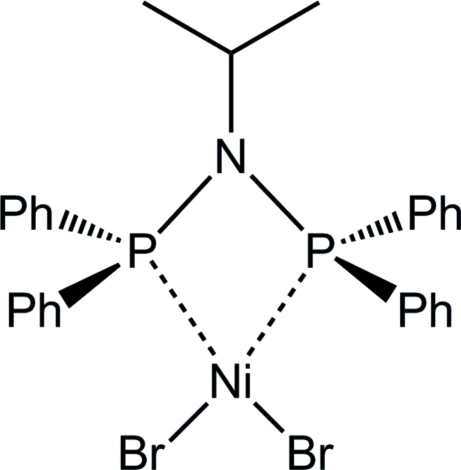


## Experimental

### 

#### Crystal data

[NiBr_2_(C_27_H_27_NP_2_)]
*M* *_r_* = 645.97
Orthorhombic, 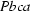

*a* = 16.6720 (3) Å
*b* = 15.1689 (4) Å
*c* = 20.3777 (4) Å
*V* = 5153.44 (19) Å^3^
*Z* = 8
Mo *K*α radiation
μ = 4.00 mm^−1^
*T* = 200 (2) K
0.17 × 0.14 × 0.04 mm

#### Data collection

Stoe IPDS-II diffractometer
Absorption correction: numerical (*X-SHAPE*; Stoe & Cie, 2005 ▶) *T*_min_ = 0.484, *T*_max_ = 0.884
73587 measured reflections
6956 independent reflections
4725 reflections with *I* > 2σ(*I*)
*R*_int_ = 0.080

#### Refinement

*R*[*F* ^2^ > 2σ(*F* ^2^)] = 0.036
*wR*(*F* ^2^) = 0.064
*S* = 0.89
6956 reflections
300 parameters
H-atom parameters constrained
Δρ_max_ = 0.57 e Å^−3^
Δρ_min_ = −0.43 e Å^−3^

### 

Data collection: *X-AREA* (Stoe & Cie, 2005 ▶); cell refinement: *X-AREA*; data reduction: *X-RED* (Stoe & Cie, 2005 ▶); program(s) used to solve structure: *SHELXS97* (Sheldrick, 2008 ▶); program(s) used to refine structure: *SHELXL97* (Sheldrick, 2008 ▶); molecular graphics: *SHELXTL* (Sheldrick, 2008 ▶); software used to prepare material for publication: *SHELXTL*.

## Supplementary Material

Crystal structure: contains datablocks I, global. DOI: 10.1107/S1600536809003936/ci2764sup1.cif

Structure factors: contains datablocks I. DOI: 10.1107/S1600536809003936/ci2764Isup2.hkl

Additional supplementary materials:  crystallographic information; 3D view; checkCIF report

## Figures and Tables

**Fig. 1.**
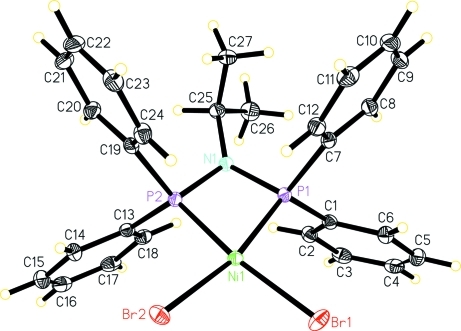
The molecular structure of the title compound showing the atom-labelling scheme. Displacement ellipsoids are drawn at the 30% probability level.

## supplementary crystallographic information

### Comment

Ligands containing the "PNP" moiety as the structural motif of the coordination unit have been used for different purposes in coordination chemistry. During the recent period, they were used with different metals including nickel for investigations into oligomerizations, polymerizations (Cooley *et al.*, 2001) or copolymerizations (Majoumo-Mbe *et al.*, 2005) with ethene or other alkenes (Sun *et al.*, 2006). The Ni(PNP) core was also used for investigations into the reactivity behaviour of the nickel-coordinated HN(PPH_2_)_2_ ligand (Sushev *et al.*, 2005). During these studies allylation of the N—H yielded a comparable nickel complex to the one that is described here. Dinuclear Ni(PNP)-complexes with arene-bridged PNP units have been prepared that have two independent and structurally identical Ni(PNP) moities (Majoumo-Mbe *et al.*, 2005).

We became interested in nickel complexes during our studies on the selective oligomerization of ethene *via* transition metal-catalyzed tri- or tetramerization, yielding 1-hexene or 1-octene (Wöhl *et al.*, 2009). Our initial experimental work was focusing on a chromium-based catalyst system (CrCl_3_(THF)_3_/Ph_2_PN(*i*Pr)PPh_2_/MAO) and we recently became interested in the kinetic behaviour of this catalyst system, to gain a better understanding of the underlying catalytic mechanism in dependence from different metal/ligand ratios. However, for reasons of comparison we wanted to examine the corresponding nickel complex containing the same simple isopropyl-substituted PNP ligand. We deployed a simple preparation procedure, that is described here, to obtain the complex in high yields for our screening experiments.

The molecular structure of the title compound shows that the Ni^II^ center is coordinated by two P atoms of the chelating Ph_2_PN(*i*Pr)PPh_2_ ligand and two bromide ions (Fig. 1). Its coordination geometry can be best described as distorted square-planar (P2—Ni1—P1 73.22 (3)°, P1—Ni1—Br1 94.39 (2)°, P2—Ni1—Br2 94.74 (2)°, Br1—Ni1—Br2 98.213 (16)°). Furthermore, the chelating ligand and the metal form a four-membered Ni(PNP) ring which is nearly planar (mean deviation from the best plane defined by Ni1, P1, N1 and P2 atoms is 0.0481 Å).

### Experimental

NiBr_2_(1,2-dimethoxyethane) (334 mg, 1.08 mmol) was dissolved in dry methanol and heated to 333 K. *N*,*N*-bis(diphenylphosphino)isopropylamine (462 mg, 1.08 mmol) was dissolved in dry THF and the solution was cannulated into the nickel complex solution under argon. The red solution obtained was stirred for an hour at 333 K and after cooling, the red solid obtained was collected on a glas frit and washed twice with methanol and three times with water and finally with ether. The red solid was dried in high vacuo to yield 645 mg of pure red complex. The identity of the product was proven by ^1^H, ^13^C and ^31^P NMR (solvent: CD_2_Cl_2_). Single crystals suitable for X-ray analysis were grown from a chloroform-diethyl ether solution (2:1).

### Refinement

All H atoms were placed in idealized positions with d(C—H) = 0.98 (CH_3_) and 0.95–1.00 Å (CH) and refined using a riding model with *U*_iso_(H) fixed at 1.5 *U*_eq_(C) for CH_3_ and 1.2 *U*_eq_(C) for CH.

### Crystal data

**Table:**

[NiBr_2_(C_27_H_27_NP_2_)]	*F*(000) = 2592
*M**_r_* = 645.97	*D*_x_ = 1.665 Mg m^−^^3^
Orthorhombic, *P**b**c**a*	Mo *K*α radiation, λ = 0.71073 Å
Hall symbol: -P 2ac 2ab	Cell parameters from 33195 reflections
*a* = 16.6720 (3) Å	θ = 2.1–29.5°
*b* = 15.1689 (4) Å	µ = 4.00 mm^−^^1^
*c* = 20.3777 (4) Å	*T* = 200 K
*V* = 5153.44 (19) Å^3^	Prism, red–brown
*Z* = 8	0.17 × 0.14 × 0.04 mm

### Data collection

**Table:**

Stoe IPDS-II diffractometer	6956 independent reflections
Radiation source: fine-focus sealed tube	4725 reflections with *I* > 2σ(*I*)
graphite	*R*_int_ = 0.080
rotation method scans	θ_max_ = 29.3°, θ_min_ = 2.0°
Absorption correction: numerical (*X-SHAPE*; Stoe & Cie, 2005)	*h* = −22→22
*T*_min_ = 0.484, *T*_max_ = 0.884	*k* = −20→20
73587 measured reflections	*l* = −27→27

### Refinement

**Table:**

Refinement on *F*^2^	Primary atom site location: structure-invariant direct methods
Least-squares matrix: full	Secondary atom site location: difference Fourier map
*R*[*F*^2^ > 2σ(*F*^2^)] = 0.036	Hydrogen site location: inferred from neighbouring sites
*wR*(*F*^2^) = 0.064	H-atom parameters constrained
*S* = 0.89	*w* = 1/[σ^2^(*F*_o_^2^) + (0.0281*P*)^2^] where *P* = (*F*_o_^2^ + 2*F*_c_^2^)/3
6956 reflections	(Δ/σ)_max_ = 0.001
300 parameters	Δρ_max_ = 0.57 e Å^−^^3^
0 restraints	Δρ_min_ = −0.43 e Å^−^^3^

### Special details

**Table:**

Geometry. All e.s.d.'s (except the e.s.d. in the dihedral angle between two l.s. planes) are estimated using the full covariance matrix. The cell e.s.d.'s are taken into account individually in the estimation of e.s.d.'s in distances, angles and torsion angles; correlations between e.s.d.'s in cell parameters are only used when they are defined by crystal symmetry. An approximate (isotropic) treatment of cell e.s.d.'s is used for estimating e.s.d.'s involving l.s. planes.
Refinement. Refinement of *F*^2^ against ALL reflections. The weighted *R*-factor *wR* and goodness of fit *S* are based on *F*^2^, conventional *R*-factors *R* are based on *F*, with *F* set to zero for negative *F*^2^. The threshold expression of *F*^2^ > σ(*F*^2^) is used only for calculating *R*-factors(gt) *etc*. and is not relevant to the choice of reflections for refinement. *R*-factors based on *F*^2^ are statistically about twice as large as those based on *F*, and *R*- factors based on ALL data will be even larger.

### Fractional atomic coordinates and isotropic or equivalent isotropic displacement parameters (Å^2^)

**Table:**

	*x*	*y*	*z*	*U*_iso_*/*U*_eq_	
C1	0.90625 (17)	0.87979 (17)	0.23217 (13)	0.0230 (5)	
C2	0.88616 (18)	0.96307 (18)	0.20951 (14)	0.0274 (6)	
H2	0.8694	0.9707	0.1653	0.033*	
C3	0.89045 (19)	1.03524 (19)	0.25112 (16)	0.0337 (7)	
H3	0.8760	1.0921	0.2357	0.040*	
C4	0.91578 (19)	1.0242 (2)	0.31503 (15)	0.0354 (7)	
H4	0.9175	1.0733	0.3439	0.043*	
C5	0.9386 (2)	0.9421 (2)	0.33713 (15)	0.0334 (7)	
H5	0.9575	0.9353	0.3808	0.040*	
C6	0.93436 (18)	0.86966 (19)	0.29619 (14)	0.0292 (6)	
H6	0.9505	0.8133	0.3116	0.035*	
C7	0.87114 (17)	0.69391 (17)	0.22892 (13)	0.0227 (5)	
C8	0.81317 (17)	0.70629 (18)	0.27757 (13)	0.0266 (6)	
H8	0.7957	0.7640	0.2886	0.032*	
C9	0.78135 (19)	0.6339 (2)	0.30950 (14)	0.0322 (7)	
H9	0.7416	0.6418	0.3424	0.039*	
C10	0.8074 (2)	0.5497 (2)	0.29349 (15)	0.0366 (7)	
H10	0.7850	0.5001	0.3153	0.044*	
C11	0.8656 (2)	0.53756 (19)	0.24623 (16)	0.0342 (7)	
H11	0.8838	0.4798	0.2359	0.041*	
C12	0.89720 (18)	0.60961 (17)	0.21390 (14)	0.0264 (6)	
H12	0.9371	0.6013	0.1812	0.032*	
C13	0.92171 (17)	0.85623 (17)	−0.00596 (13)	0.0231 (5)	
C14	0.96284 (19)	0.8386 (2)	−0.06458 (13)	0.0301 (6)	
H14	0.9826	0.7811	−0.0734	0.036*	
C15	0.9743 (2)	0.9060 (2)	−0.10958 (14)	0.0377 (7)	
H15	1.0014	0.8945	−0.1497	0.045*	
C16	0.9466 (2)	0.9895 (2)	−0.09623 (15)	0.0383 (8)	
H16	0.9554	1.0354	−0.1272	0.046*	
C17	0.90638 (19)	1.00777 (19)	−0.03885 (15)	0.0347 (7)	
H17	0.8876	1.0657	−0.0302	0.042*	
C18	0.89349 (18)	0.94077 (18)	0.00636 (15)	0.0274 (6)	
H18	0.8653	0.9528	0.0459	0.033*	
C19	0.88235 (17)	0.67305 (17)	0.01232 (13)	0.0239 (6)	
C20	0.82043 (18)	0.67961 (19)	−0.03337 (14)	0.0297 (6)	
H20	0.8025	0.7359	−0.0477	0.036*	
C21	0.7852 (2)	0.6040 (2)	−0.05781 (16)	0.0362 (7)	
H21	0.7427	0.6081	−0.0887	0.043*	
C22	0.8119 (2)	0.5222 (2)	−0.03737 (16)	0.0409 (8)	
H22	0.7867	0.4705	−0.0535	0.049*	
C23	0.8747 (2)	0.51497 (19)	0.00613 (15)	0.0368 (8)	
H23	0.8935	0.4584	0.0189	0.044*	
C24	0.9107 (2)	0.59054 (17)	0.03148 (14)	0.0295 (6)	
H24	0.9542	0.5859	0.0615	0.035*	
C25	0.75815 (16)	0.79720 (19)	0.10263 (13)	0.0269 (6)	
H25	0.7481	0.8093	0.0551	0.032*	
C26	0.71793 (19)	0.8703 (2)	0.14099 (16)	0.0382 (7)	
H26A	0.7308	0.8640	0.1877	0.057*	
H26B	0.6597	0.8668	0.1349	0.057*	
H26C	0.7373	0.9275	0.1252	0.057*	
C27	0.72121 (18)	0.7077 (2)	0.11777 (15)	0.0355 (7)	
H27A	0.7526	0.6613	0.0963	0.053*	
H27B	0.6659	0.7061	0.1015	0.053*	
H27C	0.7213	0.6980	0.1653	0.053*	
N1	0.84753 (13)	0.79808 (13)	0.11239 (10)	0.0206 (4)	
P1	0.90770 (4)	0.78459 (4)	0.17912 (3)	0.01915 (13)	
P2	0.91702 (4)	0.77028 (5)	0.05515 (3)	0.01925 (12)	
Ni1	1.013681 (19)	0.761785 (18)	0.122690 (17)	0.01868 (7)	
Br1	1.100033 (18)	0.77856 (2)	0.211279 (14)	0.03435 (8)	
Br2	1.108991 (17)	0.72404 (2)	0.043377 (14)	0.03133 (7)	

### Atomic displacement parameters (Å^2^)

**Table:**

	*U*^11^	*U*^22^	*U*^33^	*U*^12^	*U*^13^	*U*^23^
C1	0.0219 (14)	0.0241 (12)	0.0231 (12)	−0.0011 (11)	0.0044 (11)	−0.0029 (10)
C2	0.0303 (16)	0.0250 (13)	0.0270 (14)	−0.0001 (11)	0.0045 (12)	−0.0021 (11)
C3	0.0303 (16)	0.0259 (13)	0.0449 (17)	−0.0008 (12)	0.0109 (14)	−0.0037 (12)
C4	0.0292 (17)	0.0357 (16)	0.0414 (17)	−0.0099 (13)	0.0115 (14)	−0.0182 (13)
C5	0.0347 (18)	0.0394 (17)	0.0262 (14)	−0.0058 (13)	0.0022 (12)	−0.0094 (12)
C6	0.0306 (16)	0.0311 (15)	0.0258 (14)	−0.0013 (12)	0.0012 (12)	−0.0011 (11)
C7	0.0241 (14)	0.0227 (12)	0.0213 (12)	−0.0011 (10)	−0.0034 (10)	0.0042 (10)
C8	0.0259 (14)	0.0289 (14)	0.0250 (13)	0.0011 (11)	0.0007 (11)	0.0023 (10)
C9	0.0250 (16)	0.0439 (17)	0.0277 (15)	−0.0039 (13)	0.0029 (12)	0.0095 (12)
C10	0.0344 (18)	0.0400 (17)	0.0354 (16)	−0.0123 (14)	−0.0077 (14)	0.0151 (13)
C11	0.0381 (18)	0.0251 (13)	0.0394 (17)	−0.0028 (13)	−0.0101 (15)	0.0030 (13)
C12	0.0302 (16)	0.0229 (12)	0.0262 (13)	−0.0005 (11)	−0.0015 (13)	0.0016 (10)
C13	0.0204 (14)	0.0257 (13)	0.0231 (12)	−0.0043 (10)	−0.0036 (11)	0.0051 (10)
C14	0.0333 (17)	0.0350 (15)	0.0219 (13)	−0.0046 (12)	0.0019 (12)	0.0024 (11)
C15	0.0383 (19)	0.0511 (18)	0.0238 (15)	−0.0095 (14)	−0.0015 (13)	0.0090 (12)
C16	0.0344 (18)	0.0447 (18)	0.0357 (16)	−0.0137 (14)	−0.0132 (14)	0.0201 (14)
C17	0.0291 (16)	0.0302 (14)	0.0450 (18)	−0.0040 (12)	−0.0141 (15)	0.0112 (13)
C18	0.0218 (15)	0.0293 (14)	0.0313 (15)	0.0000 (11)	−0.0055 (12)	0.0017 (11)
C19	0.0256 (15)	0.0248 (13)	0.0214 (12)	−0.0031 (10)	0.0052 (11)	−0.0032 (10)
C20	0.0270 (15)	0.0336 (14)	0.0285 (14)	−0.0015 (12)	0.0010 (12)	−0.0080 (11)
C21	0.0280 (17)	0.0457 (18)	0.0349 (16)	−0.0097 (13)	0.0010 (13)	−0.0152 (14)
C22	0.042 (2)	0.0386 (17)	0.0425 (18)	−0.0196 (15)	0.0107 (16)	−0.0170 (14)
C23	0.051 (2)	0.0252 (15)	0.0342 (16)	−0.0063 (13)	0.0137 (15)	−0.0042 (12)
C24	0.0395 (18)	0.0235 (13)	0.0256 (13)	0.0011 (12)	0.0028 (13)	−0.0013 (11)
C25	0.0169 (13)	0.0370 (15)	0.0269 (13)	0.0022 (11)	−0.0018 (10)	0.0029 (11)
C26	0.0249 (16)	0.0482 (18)	0.0414 (18)	0.0097 (13)	0.0035 (13)	−0.0029 (14)
C27	0.0255 (15)	0.0466 (17)	0.0345 (15)	−0.0115 (12)	−0.0005 (13)	0.0015 (14)
N1	0.0174 (11)	0.0243 (10)	0.0200 (11)	0.0023 (8)	−0.0007 (8)	0.0002 (8)
P1	0.0200 (3)	0.0191 (3)	0.0183 (3)	0.0005 (3)	0.0009 (2)	−0.0003 (2)
P2	0.0198 (3)	0.0197 (3)	0.0183 (3)	−0.0001 (3)	−0.0003 (2)	−0.0003 (2)
Ni1	0.01720 (14)	0.01814 (14)	0.02069 (13)	0.00026 (11)	0.00008 (15)	0.00020 (13)
Br1	0.03006 (16)	0.03626 (16)	0.03674 (15)	0.00651 (14)	−0.01348 (13)	−0.00788 (13)
Br2	0.02687 (15)	0.03284 (14)	0.03429 (14)	0.00272 (13)	0.00987 (12)	−0.00076 (12)

### Geometric parameters (Å, °)

**Table:**

C1—C2	1.386 (4)	C17—C18	1.389 (4)
C1—C6	1.395 (4)	C17—H17	0.95
C1—P1	1.804 (3)	C18—H18	0.95
C2—C3	1.387 (4)	C19—C20	1.394 (4)
C2—H2	0.95	C19—C24	1.394 (4)
C3—C4	1.379 (5)	C19—P2	1.809 (3)
C3—H3	0.95	C20—C21	1.382 (4)
C4—C5	1.378 (5)	C20—H20	0.95
C4—H4	0.95	C21—C22	1.381 (5)
C5—C6	1.381 (4)	C21—H21	0.95
C5—H5	0.95	C22—C23	1.376 (5)
C6—H6	0.95	C22—H22	0.95
C7—C12	1.385 (4)	C23—C24	1.393 (4)
C7—C8	1.397 (4)	C23—H23	0.95
C7—P1	1.815 (3)	C24—H24	0.95
C8—C9	1.382 (4)	C25—N1	1.504 (3)
C8—H8	0.95	C25—C26	1.514 (4)
C9—C10	1.388 (5)	C25—C27	1.522 (4)
C9—H9	0.95	C25—H25	1.00
C10—C11	1.379 (5)	C26—H26A	0.98
C10—H10	0.95	C26—H26B	0.98
C11—C12	1.381 (4)	C26—H26C	0.98
C11—H11	0.95	C27—H27A	0.98
C12—H12	0.95	C27—H27B	0.98
C13—C18	1.389 (4)	C27—H27C	0.98
C13—C14	1.403 (4)	N1—P2	1.697 (2)
C13—P2	1.805 (3)	N1—P1	1.702 (2)
C14—C15	1.387 (4)	P1—Ni1	2.1364 (7)
C14—H14	0.95	P1—P2	2.5402 (9)
C15—C16	1.376 (5)	P2—Ni1	2.1231 (7)
C15—H15	0.95	Ni1—Br1	2.3230 (4)
C16—C17	1.376 (5)	Ni1—Br2	2.3377 (4)
C16—H16	0.95		
			
C2—C1—C6	119.6 (3)	C19—C20—H20	120.1
C2—C1—P1	122.2 (2)	C22—C21—C20	120.0 (3)
C6—C1—P1	117.9 (2)	C22—C21—H21	120.0
C1—C2—C3	120.2 (3)	C20—C21—H21	120.0
C1—C2—H2	119.9	C23—C22—C21	120.7 (3)
C3—C2—H2	119.9	C23—C22—H22	119.6
C4—C3—C2	119.8 (3)	C21—C22—H22	119.6
C4—C3—H3	120.1	C22—C23—C24	120.1 (3)
C2—C3—H3	120.1	C22—C23—H23	120.0
C5—C4—C3	120.2 (3)	C24—C23—H23	120.0
C5—C4—H4	119.9	C23—C24—C19	119.2 (3)
C3—C4—H4	119.9	C23—C24—H24	120.4
C4—C5—C6	120.5 (3)	C19—C24—H24	120.4
C4—C5—H5	119.8	N1—C25—C26	111.4 (2)
C6—C5—H5	119.8	N1—C25—C27	112.5 (2)
C5—C6—C1	119.6 (3)	C26—C25—C27	111.7 (3)
C5—C6—H6	120.2	N1—C25—H25	107.0
C1—C6—H6	120.2	C26—C25—H25	107.0
C12—C7—C8	119.9 (2)	C27—C25—H25	107.0
C12—C7—P1	118.1 (2)	C25—C26—H26A	109.5
C8—C7—P1	121.8 (2)	C25—C26—H26B	109.5
C9—C8—C7	119.5 (3)	H26A—C26—H26B	109.5
C9—C8—H8	120.2	C25—C26—H26C	109.5
C7—C8—H8	120.2	H26A—C26—H26C	109.5
C8—C9—C10	120.0 (3)	H26B—C26—H26C	109.5
C8—C9—H9	120.0	C25—C27—H27A	109.5
C10—C9—H9	120.0	C25—C27—H27B	109.5
C11—C10—C9	120.5 (3)	H27A—C27—H27B	109.5
C11—C10—H10	119.7	C25—C27—H27C	109.5
C9—C10—H10	119.7	H27A—C27—H27C	109.5
C10—C11—C12	119.7 (3)	H27B—C27—H27C	109.5
C10—C11—H11	120.1	C25—N1—P2	125.69 (17)
C12—C11—H11	120.1	C25—N1—P1	133.53 (17)
C11—C12—C7	120.4 (3)	P2—N1—P1	96.71 (11)
C11—C12—H12	119.8	N1—P1—C1	112.00 (12)
C7—C12—H12	119.8	N1—P1—C7	109.87 (12)
C18—C13—C14	119.7 (3)	C1—P1—C7	105.49 (12)
C18—C13—P2	121.8 (2)	N1—P1—Ni1	94.41 (8)
C14—C13—P2	118.1 (2)	C1—P1—Ni1	117.60 (9)
C15—C14—C13	119.3 (3)	C7—P1—Ni1	117.13 (9)
C15—C14—H14	120.3	C1—P1—P2	131.69 (9)
C13—C14—H14	120.3	C7—P1—P2	120.79 (9)
C16—C15—C14	120.1 (3)	Ni1—P1—P2	53.15 (2)
C16—C15—H15	119.9	N1—P2—C13	108.92 (12)
C14—C15—H15	119.9	N1—P2—C19	108.43 (12)
C15—C16—C17	121.1 (3)	C13—P2—C19	105.66 (13)
C15—C16—H16	119.4	N1—P2—Ni1	95.03 (8)
C17—C16—H16	119.4	C13—P2—Ni1	117.27 (9)
C16—C17—C18	119.5 (3)	C19—P2—Ni1	120.40 (10)
C16—C17—H17	120.3	C13—P2—P1	128.84 (9)
C18—C17—H17	120.3	C19—P2—P1	122.01 (9)
C17—C18—C13	120.2 (3)	Ni1—P2—P1	53.63 (2)
C17—C18—H18	119.9	P2—Ni1—P1	73.22 (3)
C13—C18—H18	119.9	P2—Ni1—Br1	165.35 (2)
C20—C19—C24	120.2 (3)	P1—Ni1—Br1	94.39 (2)
C20—C19—P2	120.1 (2)	P2—Ni1—Br2	94.74 (2)
C24—C19—P2	119.2 (2)	P1—Ni1—Br2	166.90 (3)
C21—C20—C19	119.7 (3)	Br1—Ni1—Br2	98.213 (16)
C21—C20—H20	120.1		
			
C6—C1—C2—C3	−3.0 (4)	C25—N1—P2—C13	72.5 (2)
P1—C1—C2—C3	−176.2 (2)	P1—N1—P2—C13	−127.71 (12)
C1—C2—C3—C4	0.8 (4)	C25—N1—P2—C19	−42.0 (2)
C2—C3—C4—C5	1.6 (5)	P1—N1—P2—C19	117.77 (12)
C3—C4—C5—C6	−1.8 (5)	C25—N1—P2—Ni1	−166.4 (2)
C4—C5—C6—C1	−0.4 (5)	P1—N1—P2—Ni1	−6.62 (9)
C2—C1—C6—C5	2.7 (4)	C25—N1—P2—P1	−159.8 (3)
P1—C1—C6—C5	176.2 (2)	C18—C13—P2—N1	19.8 (3)
C12—C7—C8—C9	−1.1 (4)	C14—C13—P2—N1	−167.1 (2)
P1—C7—C8—C9	173.4 (2)	C18—C13—P2—C19	136.1 (2)
C7—C8—C9—C10	0.4 (4)	C14—C13—P2—C19	−50.8 (3)
C8—C9—C10—C11	0.6 (5)	C18—C13—P2—Ni1	−86.5 (2)
C9—C10—C11—C12	−1.0 (5)	C14—C13—P2—Ni1	86.6 (2)
C10—C11—C12—C7	0.3 (5)	C18—C13—P2—P1	−22.7 (3)
C8—C7—C12—C11	0.7 (4)	C14—C13—P2—P1	150.41 (18)
P1—C7—C12—C11	−174.0 (2)	C20—C19—P2—N1	74.3 (2)
C18—C13—C14—C15	−0.3 (4)	C24—C19—P2—N1	−97.5 (2)
P2—C13—C14—C15	−173.6 (2)	C20—C19—P2—C13	−42.4 (3)
C13—C14—C15—C16	0.9 (5)	C24—C19—P2—C13	145.9 (2)
C14—C15—C16—C17	−0.8 (5)	C20—C19—P2—Ni1	−178.12 (19)
C15—C16—C17—C18	0.0 (5)	C24—C19—P2—Ni1	10.1 (3)
C16—C17—C18—C13	0.7 (4)	C20—C19—P2—P1	118.2 (2)
C14—C13—C18—C17	−0.5 (4)	C24—C19—P2—P1	−53.5 (3)
P2—C13—C18—C17	172.5 (2)	C1—P1—P2—N1	−75.63 (17)
C24—C19—C20—C21	2.4 (4)	C7—P1—P2—N1	85.67 (15)
P2—C19—C20—C21	−169.3 (2)	Ni1—P1—P2—N1	−171.81 (11)
C19—C20—C21—C22	−0.6 (5)	N1—P1—P2—C13	73.89 (16)
C20—C21—C22—C23	−1.5 (5)	C1—P1—P2—C13	−1.73 (18)
C21—C22—C23—C24	1.8 (5)	C7—P1—P2—C13	159.56 (16)
C22—C23—C24—C19	0.1 (5)	Ni1—P1—P2—C13	−97.92 (12)
C20—C19—C24—C23	−2.2 (4)	N1—P1—P2—C19	−81.89 (16)
P2—C19—C24—C23	169.6 (2)	C1—P1—P2—C19	−157.52 (17)
C26—C25—N1—P2	−146.0 (2)	C7—P1—P2—C19	3.78 (16)
C27—C25—N1—P2	87.8 (3)	Ni1—P1—P2—C19	106.30 (12)
C26—C25—N1—P1	62.3 (3)	N1—P1—P2—Ni1	171.81 (11)
C27—C25—N1—P1	−64.0 (3)	C1—P1—P2—Ni1	96.18 (13)
C25—N1—P1—C1	−74.0 (3)	C7—P1—P2—Ni1	−102.52 (11)
P2—N1—P1—C1	128.73 (12)	N1—P2—Ni1—P1	5.46 (7)
C25—N1—P1—C7	42.9 (3)	C13—P2—Ni1—P1	119.77 (11)
P2—N1—P1—C7	−114.38 (12)	C19—P2—Ni1—P1	−109.35 (10)
C25—N1—P1—Ni1	163.8 (2)	N1—P2—Ni1—Br1	−27.65 (13)
P2—N1—P1—Ni1	6.57 (9)	C13—P2—Ni1—Br1	86.66 (14)
C25—N1—P1—P2	157.2 (3)	C19—P2—Ni1—Br1	−142.46 (12)
C2—C1—P1—N1	−23.8 (3)	P1—P2—Ni1—Br1	−33.12 (10)
C6—C1—P1—N1	162.9 (2)	N1—P2—Ni1—Br2	−179.80 (7)
C2—C1—P1—C7	−143.3 (2)	C13—P2—Ni1—Br2	−65.49 (11)
C6—C1—P1—C7	43.4 (3)	C19—P2—Ni1—Br2	65.39 (10)
C2—C1—P1—Ni1	84.0 (3)	P1—P2—Ni1—Br2	174.74 (2)
C6—C1—P1—Ni1	−89.3 (2)	N1—P1—Ni1—P2	−5.44 (7)
C2—C1—P1—P2	20.1 (3)	C1—P1—Ni1—P2	−123.10 (10)
C6—C1—P1—P2	−153.19 (18)	C7—P1—Ni1—P2	109.55 (10)
C12—C7—P1—N1	87.2 (2)	N1—P1—Ni1—Br1	166.59 (7)
C8—C7—P1—N1	−87.4 (2)	C1—P1—Ni1—Br1	48.93 (10)
C12—C7—P1—C1	−151.9 (2)	C7—P1—Ni1—Br1	−78.41 (10)
C8—C7—P1—C1	33.5 (3)	P2—P1—Ni1—Br1	172.04 (2)
C12—C7—P1—Ni1	−18.9 (3)	N1—P1—Ni1—Br2	−29.24 (14)
C8—C7—P1—Ni1	166.51 (19)	C1—P1—Ni1—Br2	−146.90 (13)
C12—C7—P1—P2	42.5 (3)	C7—P1—Ni1—Br2	85.76 (14)
C8—C7—P1—P2	−132.1 (2)	P2—P1—Ni1—Br2	−23.79 (11)
